# Resveratrol and vascular health: evidence from clinical studies and mechanisms of actions related to its metabolites produced by gut microbiota

**DOI:** 10.3389/fphar.2024.1368949

**Published:** 2024-03-18

**Authors:** Justyna Godos, Giovanni Luca Romano, Lucia Gozzo, Samuele Laudani, Nadia Paladino, Irma Dominguez Azpíroz, Nohora Milena Martínez López, Francesca Giampieri, José L. Quiles, Maurizio Battino, Fabio Galvano, Filippo Drago, Giuseppe Grosso

**Affiliations:** ^1^ Department of Biomedical and Biotechnological Sciences, University of Catania, Catania, Italy; ^2^ Department of Medicine and Surgery, University of Enna “Kore”, Enna, Italy; ^3^ Clinical Pharmacology Unit/Regional Pharmacovigilance Centre, Azienda Ospedaliero Universitaria Policlinico “G. Rodolico-S. Marco”, Catania, Italy; ^4^ Research Group on Food, Nutritional Biochemistry and Health, Universidad Europea del Atlántico, Santander, Spain; ^5^ Universidade Internacional do Cuanza, Cuito, Angola; ^6^ Universidad de La Romana, La Romana, Dominican Republic; ^7^ Universidad Internacional Iberoamericana, Campeche, Mexico; ^8^ Fundación Universitaria Internacional de Colombia, Bogotá, Colombia; ^9^ Department of Clinical Sciences, Università Politecnica delle Marche, Ancona, Italy; ^10^ Department of Physiology, Institute of Nutrition and Food Technology “José Mataix”, Biomedical Research Center, University of Granada, Parque Tecnologico de la Salud, Granada, Spain; ^11^ Research and Development Functional Food Centre (CIDAF), Health Science Technological Park, Granada, Spain; ^12^ International Joint Research Laboratory of Intelligent Agriculture and Agri-products Processing, Jiangsu University, Zhenjiang, Jiangsu, China; ^13^ Center for Human Nutrition and Mediterranean Foods (NUTREA), University of Catania, Catania, Italy

**Keywords:** resveratrol, polyphenols, metabolites, vascular, gut microbiota

## Abstract

Cardiovascular diseases are among the leading causes of mortality worldwide, with dietary factors being the main risk contributors. Diets rich in bioactive compounds, such as (poly)phenols, have been shown to potentially exert positive effects on vascular health. Among them, resveratrol has gained particular attention due to its potential antioxidant and anti-inflammatory action. Nevertheless, the results in humans are conflicting possibly due to interindividual different responses. The gut microbiota, a complex microbial community that inhabits the gastrointestinal tract, has been called out as potentially responsible for modulating the biological activities of phenolic metabolites in humans. The present review aims to summarize the main findings from clinical trials on the effects of resveratrol interventions on endothelial and vascular outcomes and review potential mechanisms interesting the role of gut microbiota on the metabolism of this molecule and its cardioprotective metabolites. The findings from randomized controlled trials show contrasting results on the effects of resveratrol supplementation and vascular biomarkers without dose-dependent effect. In particular, studies in which resveratrol was integrated using food sources, i.e., red wine, reported significant effects although the resveratrol content was, on average, much lower compared to tablet supplementation, while other studies with often extreme resveratrol supplementation resulted in null findings. The results from experimental studies suggest that resveratrol exerts cardioprotective effects through the modulation of various antioxidant, anti-inflammatory, and anti-hypertensive pathways, and microbiota composition. Recent studies on resveratrol-derived metabolites, such as piceatannol, have demonstrated its effects on biomarkers of vascular health. Moreover, resveratrol itself has been shown to improve the gut microbiota composition toward an anti-inflammatory profile. Considering the contrasting findings from clinical studies, future research exploring the bidirectional link between resveratrol metabolism and gut microbiota as well as the mediating effect of gut microbiota in resveratrol effect on cardiovascular health is warranted.

## 1 Introduction

Cardiovascular disease (CVD) is the leading cause of death in the world and it has been estimated to cause more than 23.6 million deaths by 2030 (GBD, 2019 Diseases and Injuries Collaborators, 2020). Hypertension is one of the strongest risk factors for most cardiovascular outcomes alongside obesity and other metabolic abnormalities (Oliveras and de la Sierra, 2014). Thus, it is important to understand the pathogenic mechanisms as well as the effective strategy to prevent and manage cardiovascular-related disorders (Golia et al., 2014). Vascular and systemic inflammation seems to represent the culprit for the establishment of endothelial dysfunction (Goswami et al., 2021). Several inflammatory pathways, such as protein kinase B (PKB/Akt), transcription factor nuclear factor-kappa B (NF-κB), mitogen-activated protein kinase p38, and extracellular signal-regulated kinases (ERK)1/2 may alter the functionality of nitric oxide synthase (NOS) and lead to abnormal expression of adhesion molecules, such as intercellular adhesion molecule-1 (ICAM-1) and vascular cell adhesion protein-1 (VCAM-1) (Figueiredo et al., 2023).

Several risk factors, such as genetics, environmental and dietary factors may play a role as immune modulators and be involved in CVD onset (GBD, 2019 Risk Factors Collaborators, 2020). Concerning dietary factors, plant-based dietary patterns have been shown to be associated with a lower risk of CVD (Angelino et al., 2019; Tieri et al., 2020; Martini et al., 2021). Among the many components of plant-based dietary patterns, fruits and vegetables are rich in bioactive compounds, such as (poly)phenols, that have been demonstrated to potentially exert health benefits on the cardiovascular system (Micek et al., 2021; Laudani et al., 2023). (Poly)phenols are characterized by a great variety of chemical structures, some of them responsible for their putative effects in humans (Tsao, 2010), through the regulation of oxidative stress (Arrigoni et al., 2023), inflammation (Jantan et al., 2021), and gut microbiota (Iqbal et al., 2022). Extensive epidemiological data support the notion that a diet rich in (poly)phenol-containing fruits, vegetables, cocoa, and beverages offers protection against the onset of CVD and type 2 diabetes (Grosso et al., 2017; Angelino et al., 2019; Veronese et al., 2019).

Among the most studied compounds, resveratrol has gained great interest in research over the last few decades (Pyo et al., 2020; Repossi et al., 2020). Resveratrol is a low-molecular-weight polyphenolic compound belonging to the stilbenoid family, which consists of hydroxylated derivatives of stilbene present in a variety of plant sources like grapes and berries, as well as in peanuts and red wine (Tian and Liu, 2020). This molecule has been widely studied because of its antioxidant and anti-inflammatory activities as well as potential protective effects against different diseases, such as cancer, cardiovascular, metabolic and neurodegenerative diseases (Baur and Sinclair, 2006; Li et al., 2012; Springer and Moco, 2019). Although extensively studied in both *in vitro* and *in vivo* models, the evidence on its potential effects in humans is not univocal (Khorshidi et al., 2021). Due to its hydrophobic properties and low plasma bioavailability, there is some skepticism concerning its real efficacy in humans, while studies focusing on the role of gut microbiota in its transformation, absorption, and more bioavailable metabolites production may provide the rationale to explain the interindividual responses and the consequent heterogeneity of results from clinical trials (Man et al., 2020). The aim of this study was to review the evidence concerning the effects of resveratrol on vascular outcomes: specifically, the article provides (i) an overview of existing RCTs on resveratrol supplementation and vascular and endothelial outcomes; (ii) a summary of potential molecular mechanisms through which resveratrol may exert its effects; and (iii) a discussion the effects of resveratrol-gut microbiota derived metabolites on the such outcomes as new potential mechanisms related to gut microbiota.

## 2 Clinical studies on resveratrol and vascular outcomes

A summary of randomized controlled trials (RCTs) with resveratrol supplementation for vascular outcomes is presented in Table 1. Among clinical intervention studies administering higher doses of resveratrol through tablets or capsules, an open-label, controlled, RCT involving 57 patients with type 2 diabetes mellitus (aged between 30 and 70 years) treated with oral hypoglycemic agents and 250 mg/day of resveratrol (intervention group) or only with oral hypoglycemic agents (control group) for 6 months, revealed a significant reduction in SBP after resveratrol supplementation compared to baseline (139.71 ± 16.10 vs. 131.14 ± 9.86 mmHg; *p* = 0.01) and a significant reduction of SBP (4.31 ± 12.26 mmHg vs −8.57 ± 17.29 mmHg, *p* = 0.008) and DBP (6.20 ± 8.90 mmHg vs 0.85 ± 9.71 mmHg, *p* = 0.02) comparing treatment group to control (Bhatt and Nanjan, 2013). Similarly, another double-blind, parallel RCT investigated the effects of 1 g/day of resveratrol capsules compared with placebo in 66 patients with type 2 diabetes mellitus (mean age of 52 years). After 45 days of treatment, the intervention group showed a significant reduction in SBP compared to the baseline values (from 129.03 ± 14.91 mmHg to 121.45 ± 10.26 mmHg; *p* < 0.0001), as well as a significant reduction compared to control group (1.37 ± 4.98 mmHg vs −7.58 ± 8.04 mmHg, *p* < 0.0001) (Movahed et al., 2013). In a double-blind, crossover RCT, 11 healthy obese men (mean age of 52 years) were supplement for 30 days with 150 mg/day of resveratrol: at the end of the treatment, results revealed a significant reduction in mean arterial pressure (94.9 ± 2.9 v. s 97.9 ± 2.7 mmHg; *p* = 0.02) and in SBP (124.7 ± 3.1 vs. 130.5 ± 2.7 mmHg; *p* = 0.006) after resveratrol supplementation compared to placebo (Timmers et al., 2011), while no significant changes in DBP were observed. Another 12-week double-blind crossover RCT compared the effects of resveratrol capsules (providing 75 mg trans-resveratrol) or placebo on 28 healthy obese adults aged between 40 and 75 years: at the end of the study, a relative increase of 23% in FMD was reported compared to baseline levels (95% CI: 0.22, 2.54; *p* = 0.021) but no significant changes in BP after daily resveratrol treatment (*p* > 0.05) were observed (Wong et al., 2013). A double-blind, crossover RCT included 45 overweight and obese subjects (mean age 61 years) supplemented with 150 mg of resveratrol or a placebo for 4 weeks, spaced by a 4-week washout period: at the end of the trial, DBP (84 ± 9 mmHg vs. 86 ± 9 mmHg; *p* = 0.044) and heart rate (64 ± 8 BPM vs. 67 ± 8 BPM; *p* = 0.025) increased significantly in the resveratrol supplementation group but no significant changes were reported in SBP when comparing to baseline values, also no changes in other endothelial markers were observed between the groups (van der Made et al., 2015). Another double-blind, crossover RCT tested the effects of 150 mg/day of resveratrol in 17 patients with type-2 diabetes mellitus (40–70 years) leading to a significant reduction in left ventricular end systolic diameter (*p* = 0.04). Although a tendency in SBP reduction (*p* = 0.09) was observed after resveratrol supplementation, no changes in DBP were noted (Timmers et al., 2016). A double-blind, placebo-controlled RCT including 50 patients with type-2 diabetes mellitus (mean age 58 years) supplemented with 100 mg/day of resveratrol for 12 weeks, reported a decrease in SBP (−5.5 ± 13.0 mmHg; *p* < 0.05) and in cardio-ankle vascular index (CAVI) (−0.4 ± 0.7; *p* < 0.05) in the intervention group when compared end of trial to baseline values. Although a significant decrease in CAVI (*p* < 0.01) was observed comparing intervention group with control was observed, no significant differences between the groups were noted for SBP and DBP (Imamura et al., 2017). A double-blind placebo-controlled RCT was conducted on 45 subjects with type-2 diabetes mellitus to investigate the daily intake of 800 mg of resveratrol or placebo capsules for 8 weeks showed a significant decrease in SBP (−10.42 ± 8.40 mmHg vs. −1.475 ± 8.72 mmHg; *p* = 0.002) and DBP (−5.6 ± 6.50 mmHg vs. 1.50 ± 8.75 mmHg; *p* = 0.006) in the resveratrol group compared to the placebo group (Khodabandehloo et al., 2018). In a double-blind, placebo-controlled, RCT 46 patients with type-2 diabetes mellitus (aged between 30 and 70 years) were recruited to evaluate the effects of 2-month supplementation of 800 mg/day of resveratrol reporting a significant reduction in SBP (*p* = 0.000) and DBP (*p* = 0.000) in the intervention group when comparing end of trial results to baseline. Also, a significant reduction in SBP (−10.2 ± 8.5 vs. −1.3 ± 10.8 mmHg, *p* = 0.002) and DBP (−7.3 ± 6.8 vs. 1.1 ± 9.0 mmHg, *p* = 0.000) when comparing intervention group to placebo was observed (Seyyedebrahimi et al., 2018). Another double-blind, RCT recruited 50 patients with non-alcoholic fatty liver disease (18 years and older) to test resveratrol supplementation (a capsule a day of 500 mg of pure trans-resveratrol) for 12 weeks on BP leading to no significant changes in BP, although changes in SBP significantly differed between the intervention and the control group (Faghihzadeh et al., 2015).

**TABLE 1:** Main characteristics of the randomized controlled trials evaluating the effects of resveratrol supplementation on cardiovascular risk factors.

Author, year of publication, country	Study design	Population characteristics	Intervention duration	Intervention type	Resveratrol dose (daily intake)	Control type	Main findings
Timmers et al. (2011), Netherlands	Double-blind, placebo-controlled, crossover	11 healthy obese men (52 years)	2 × 30 days (4 weeks washout)	Resveratrol capsules	150 mg resveratrol	Placebo capsule	SBP (*p* = 0.006) and mean arterial BP (*p* = 0.02) decreased significantly after resveratrol supplementation, when compared to placebo. However, no significant changes were observed for DBP.
Bhatt and Nanjan (2013), India	Open-label, controlled	57 patients with T2DM (50 years)	6 months	Resveratrol capsules (+ hypoglycemic agent)	250 mg resveratrol	Hypoglycemic agent	SBP decreased significantly after the intervention period (*p* = 0.01) in participants who received resveratrol. SBP (*p* = 0.008) and DBP (*p* = 0.02) decreased in the intervention group compared to the control
Movahed et al. (2013), Iran	Double-blind, placebo-controlled	66 patients with T2DM (52 years)	45 days	Resveratrol capsules	1,000 mg resveratrol	Placebo capsules (inert microcellulose)	SBP significantly decreased (*p* < 0.0001) after resveratrol supplementation. Similarly, SBP decreased (*p* < 0.0001) significantly when comparing intervention group to control
Wong et al. (2013), Australia	Double-blind, placebo-controlled, crossover	28 healthy obese adults (61 years)	2 × 6 weeks	Resveratrol capsules	75 mg trans-resveratrol	Placebo capsules	Intervention led to a significant increase in FMD (*p* = 0.021), when compared to placebo
Anton et al. (2014), United States of America	Double-blind, placebo-controlled	32 overweight older adults (73 years)	90 days	Resveratrol capsules	(i) 1,000 mg resveratrol; (ii) 300 mg resveratrol	Placebo capsules (microcrystalline cellulose)	No significant changes in BP were observed
Faghihzadeh et al. (2015), Iran	Double-blind, placebo-controlled	50 patients with NAFLD (resveratrol group: 44 years; placebo group: 46 years)	12 weeks	Resveratrol capsules	500 mg resveratrol	Placebo capsules (edible paraffin)	BP did not change in the pre-post treatment, however changes in SBP significantly differed between the intervention and the control group
Van der Made et al. (2015), Netherlands	Double-blind, placebo-controlled, crossover	45 overweight and obese individuals (61 years)	2 × 4 weeks (4 weeks washout)	Resveratrol capsules	150 mg resveratrol	Placebo capsules	A significant increase in DBP (*p* = 0.044) and HR (*p* = 0.0.025) was detected after resveratrol supplementation, but no changes were observed in SBP comparing end of trial to baseline. No significant changes in other endothelial function markers were reported
Bo et al. (2016), Italy	Double-blind, placebo-controlled	192 patients T2DM (65 years)	6 months	Resveratrol capsules	(i) 500 mg resveratrol; (ii) 40 mg resveratrol	Placebo capsules (inert microcellulose)	No significant changes were found in BP after the intervention, when comparing to control
Timmers et al. (2016), Netherlands	Double-blind, placebo-controlled, crossover	17 patients with T2DM (55 years)	2 × 30 days (30 days washout)	Resveratrol capsules	150 mg resveratrol	Placebo capsules	Although a tendency in SBP reduction (*p* = 0.09) was observed after resveratrol supplementation, no changes in DBP were noted. Echocardiography revealed a marginal reduction in left ventricular end systolic diameter after resveratrol intervention (*p* = 0.04)
Imamura et al. (2016), Japan	Double-blind, placebo-controlled	50 patients with T2DM (∼58 years)	12 weeks	Resveratrol tablet	100 mg resveratrol (oligo-stilbene 27.97 mg)	Placebo tablet	After resveratrol supplementation, SBP and CAVI (*p* < 0.05) decreased significantly. CAVI decrease (*p* < 0.01) was observed also when comparing treatment group with control. However, no significant changes in SBP and DBP were observed, when comparing intervention group to control
Kjaer et al. (2017), Denmark	Double-blind, placebo-controlled	66 middle-aged community-dwelling men (49 years)	16 weeks	Resveratrol tablet	(i) 1,000 mg resveratrol; (ii) 150 mg resveratrol	Placebo tablet	No significant effects on BP were observed after resveratrol supplementation
Khodabandehloo et al. (2018), Iran	Double-blind, placebo-controlled	45 subjects with T2DM (resveratrol group: 56 years; placebo group: 61 years)	8 weeks	Resveratrol capsules	800 mg resveratrol	Placebo capsules (inert microcellulose)	SBP (*p* < 0.001) and DBP (*p* = 0.001) decreased significantly in the intervention group, when comparing end of trial to baseline. Also, a significant reduction in SBP (*p* = 0.002) and DBP (*p* = 0.006) was found in the resveratrol group compared to the placebo group
Seyyedebrahimi et al. (2018), Iran	Double-blind, placebo-controlled	46 patients with T2DM (50 years)	2 months	Resveratrol capsule	800 mg resveratrol	Placebo capsules (microcellulose)	A significant reduction in SBP (*p* = 0.000) and DBP (*p* = 0.000) was observed in the intervention group after the treatment compared to baseline. Also, a significant reduction in DBP (*p* = 0.000) and SBP (*p* = 0.002) was observed comparing intervention and placebo groups

Abbreviations: BP, blood pressure; CAVI, cardio-ankle vascular index; d, day; DBP, diastolic blood pressure; FMD, flow-mediated dilatation; HR, heart rate; mo, month; NAFLD, non-alcoholic fatty liver disease; RCT, randomized controlled trial; RGC, red grape cell powder; SBP, systolic blood pressure; T2DM, type 2 diabetes mellitus; wk, week; y, year.

However, another group of studies with similar investigation design led to null results. A 90-day double-blind, placebo-controlled, RCT investigated the effects of resveratrol in a group of 32 overweight older adults (65 years or older) randomized into three groups: (i) 1,000 mg/day of resveratrol (high dose), (ii) 300 mg/day of resveratrol (moderate dose), or (iii) placebo: after the treatment period, no significant results were reported in terms of SBP and DBP either for end of trial *versus* baseline value comparison or between the groups comparison (Anton et al., 2014). A 6-month double-blind, RCT 192 patients with type 2 diabetes mellitus (mean age about 65 years) were involved and supplemented with capsules containing different doses of resveratrol (500 mg/day or 40 mg/day) or with a placebo: at the end of the study, no significant results were reported in terms of BP improvement (Bo et al., 2016). Finally, a double-blind, parallel RCT investigated the effects of resveratrol supplementation (1,000 mg of resveratrol, 150 mg of resveratrol, or placebo tablets) in 66 middle-aged community-dwelling men (mean age 49 years) for 16 weeks: at the end of the trial, the results showed no significant differences in SBP and DBP after resveratrol treatment (Kjær et al., 2017).

## 3 Molecular mechanisms in vascular health and disease

The endothelium is a cellular monolayer covering the blood vessel wall which is important in maintaining organ health and homeostasis. Endothelium exerts numerous functions spacing from the maintenance of vascular tone to the supply of antioxidant, antithrombotic, and anti-inflammatory interfaces (Xu et al., 2021). Nitric oxide (NO) is the endothelium-relaxing derived factor produced by L-arginine from the endothelium nitric oxide synthase (eNOS) that uses tetrahydrobiopterin (BH4) as a cofactor (Förstermann and Münzel, 2006). The production of NO is regulated by different mechanisms that respond to mechanosensors/mechanosensitive complexes on the surface of endothelial cells (Chatterjee, 2018). The endothelium produces also vasoconstrictor molecules such as endothelin-1 (ET-1), angiotensin II (Ang-II), thromboxane A2 (TxA2), thrombin, and other molecules involved in many other functions such as coagulation, and platelet activity (Miller, 2006; Sharma et al., 2018). Endothelium integrity is essential to maintain the semipermeable barrier between the vascular smooth muscle and the vascular lumen (Abdelsalam et al., 2019). Different microstructures have been identified as essential for endothelial cell integrity that together are known as endothelial glycocalyx (Harding et al., 2019) that is important in regulating endothelial function such as the flow-dependent NO synthesis (Ebong et al., 2014; Harding et al., 2018), and regulate endothelial permeability (Singh et al., 2007). Different studies demonstrate that glycocalyx alteration led to increased permeability (Puerta-Guardo et al., 2019; Biering et al., 2021) and a reduction in NO synthesis (Kang et al., 2020).

**FIGURE 1:**
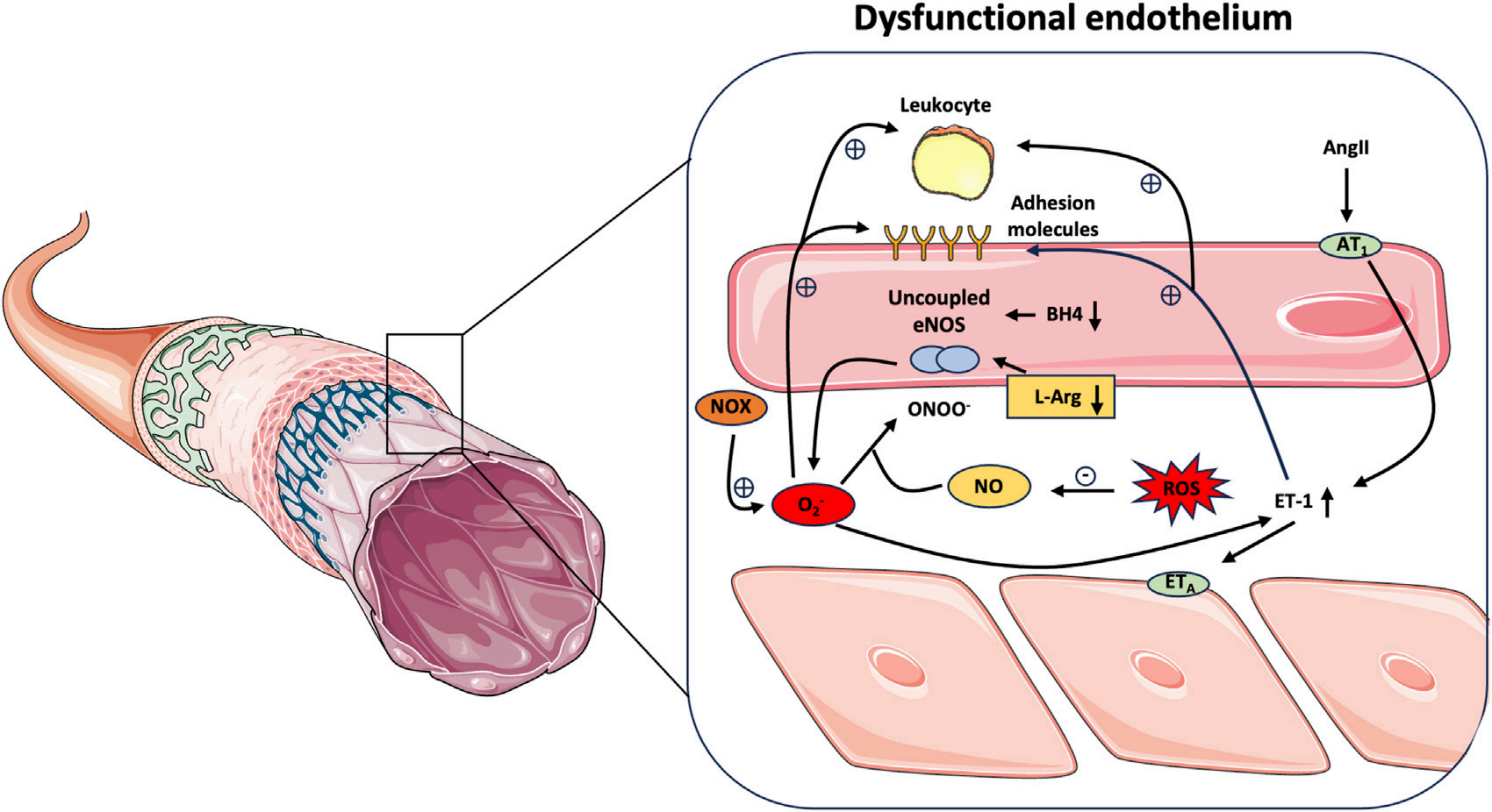
An overview of endothelial dysfunction. Different oxidative factors can contribute to eNOS uncoupling leading to the production of ROS and the reduction of NO bioavailability. Abbreviations; Ang-II, Angiotensin II; AT1, angiotensin one receptor; BH4, tetrahydrobiopterin; ET1, endothelin-1; ETA, endothelin A receptor; NO, nitric oxide; NOX, nicotinamide adenine dinucleotide phosphate oxidase; O2-, superoxide anion; ONOO-, peroxynitrite; ROS, reactive oxygen species.

Endothelial dysfunction linked to oxidative stress, inflammation, and correlated damages is the main cause of CVD onset (Figure 1). Oxidative stress can be induced by exposure to different factors such as oxLDL (Gradinaru et al., 2015), high plasma glucose, free fatty acids (Sun et al., 2019), trimethylamine-N-oxide (TMAO) (Piotrowska et al., 2018; Brunt et al., 2020), and other agents (Yan et al., 2017; Mongiardi et al., 2019). Reactive oxygen species (ROS) are produced by different enzymes like xanthine oxidase, NADPH oxidases, dysfunctional mitochondria, and uncoupled eNOS (Schulz et al., 2014). eNOS is well known for its role in the production of NO from L-arginine. However, uncoupled eNOS switch to the production of superoxide anion (O2-) (Karbach et al., 2014) that not only causes a reduction of NO production but also superoxide anion can react with NO forming peroxynitrite anion which further reduces the bioavailability of NO contributing to endothelial dysfunction (Xu et al., 2016; Daiber and Chlopicki, 2020). Different factors can contribute to eNOS uncoupling including L-arginine and BH4 deficiency, oxidative disruption of the zinc-sulfur complex (ZnCys4) of the eNOS dimer, S-glutathionylation of eNOS, and phosphorylation of eNOS at Thr495 and Tyr657 (Daiber et al., 2019; Wu et al., 2021). Another factor involved in endothelial dysfunction is inflammation. Endothelial inflammation plays a pivotal role in the progression of atherosclerosis and CVD (Haybar et al., 2019) leading to the production of proinflammatory mediators such as interleukin (IL)-8, chemokines, monocyte chemoattractant protein-1 (MCP-1), intercellular adhesion molecule-1 (ICAM-1), P-selectin, E-selectin, vascular adhesion molecule-1 (VCAM-1), and other inflammatory factors that attract monocyte and neutrophils which penetrate the arterial wall initiating the inflammatory process of atherogenesis (Chistiakov et al., 2018). The transcriptional factor NF-kB is strongly implicated in vascular inflammation by increasing proinflammatory factors such as TNF-a, IL-6, MCP-1, and IL-1b (Zhang et al., 2016). Endothelial-to-mesenchymal transition (EndoMT) is another factor implicated in endothelial dysfunction. It is characterized by the loss of endothelial morphology and the acquisition of a mesenchymal-like morphology accompanied by gene expression patterns (Chen and Simons, 2016) that involve TGF-beta. TGF-beta activation leads to the expression of transcription factors such as zinc finger E-box homeobox 1, Smads, Snail, and Slug promoting the expression of mesenchymal markers like smooth muscle protein 22a, a-SMA, collagen 1A1, vimentin, fibronectin, matrix metalloproteinase (MMP)-2, MMP-9, and FSP1 (Gonzalez and Medici, 2014; Pérez et al., 2017). EndoMT is driven by different factors such as hypoxia, chronic inflammation, oxidized lipids, hyperglycemia, and ROS production (Evrard et al., 2016) and could be considered as a link between atherosclerosis initiating factors and disturbed blood flow and plaque formation (Chen and Simons, 2016).

## 4 Potential pharmacological effects of resveratrol and mechanisms of action

**FIGURE 2:**
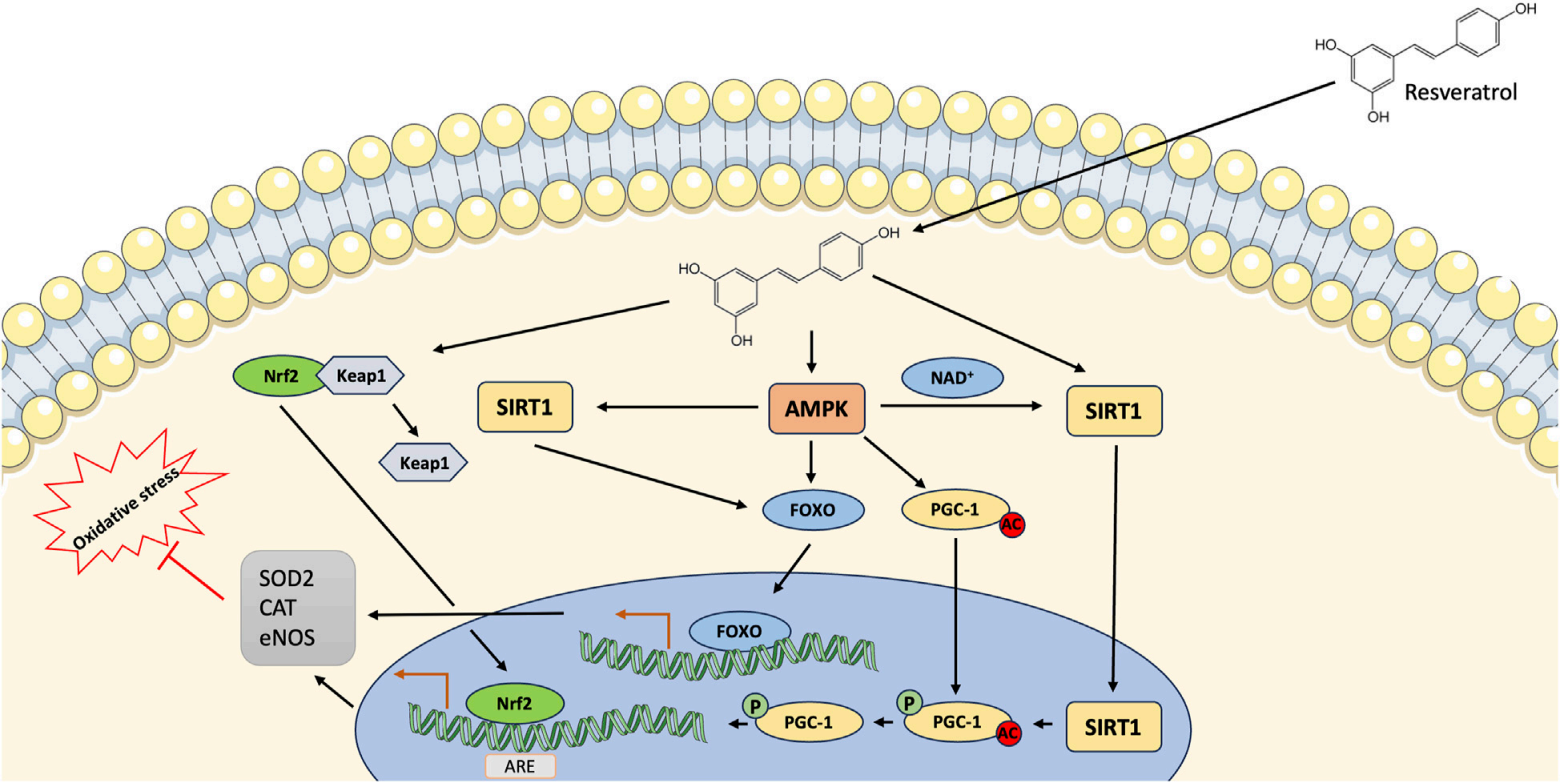
Main mechanisms by which resveratrol exerts its cardioprotective effects. Resveratrol activates AMPK/Sirt1/PGC-1 pathway resulting in the deacetylation/phosphorylation of PGC-1. As a coactivator, PGC-1 results in the activation of downstream genes, comprising multiple genes involved in regulation of mitochondrial function. AMPK and SIRT1 activation results in the nuclear translocation of FOXO and upregulation of eNOS. Resveratrol modulates the Nrf2/KEAP1 pathway through Nrf2 dissociation from KEAP1 and its translocation into nucleus that in turn activates ARE, which modulates the transcription of antioxidant enzymes. AMPK, AMP kinase; ARE, antioxidant response element; CAT, catalase; eNOS, endothelial nitric oxide synthase; FOXO, Forkhead box O; Keap1, Kelch-like ECH-associated protein 1; NAD, nicotinamide adenine dinucleotide; Nrf2, nuclear factor (erythroid-derived 2)-like 2; PGC-1, peroxisome proliferator-activated receptor gamma coactivator 1; SIRT1, sirtuin 1; SOD2, superoxide dismutase 2.

Resveratrol is largely known for its antioxidant activity. *In vitro* studies demonstrated that this (poly)phenol can directly scavenge a variety of oxidants, including hydroxyl radical, superoxide, and hydrogen peroxide (Xia et al., 2017). Resveratrol treatment showed to improve the levels of glutathione (GSH), glutathione reductase (GR), superoxide dismutase (SOD), catalase (CAT), and acetylcholinesterase (AchE) (Ibrahim et al., 2022) as well as a 14-fold increase of SOD function that, by reducing superoxide, restores mitochondrial function (Diaz-Gerevini et al., 2016) (Figure 2). Due to the hydrophobic properties of resveratrol, it is likely that its activity is mediated by binding to hydrophobic pockets in proteins. There are around 20 proteins that have been identified to interact directly with resveratrol (Britton et al., 2015). Among them, an important target of resveratrol is a particular subpopulation of estrogen receptor alpha (ER-α) associated with caveolae in the endothelial plasma membrane and coupled with eNOS via G protein (Wyckoff et al., 2001). Another important target of resveratrol is the protein sirtuin 1 (SIRT1). The cardioprotective effects of resveratrol have been historically attributed, as for many other (poly)phenol compounds, to its reactive oxygen species (ROS) scavenger activity (Xia et al., 2017). Resveratrol can increase nitric oxide (NO) bioavailability through direct ROS scavenging via Akt/endothelial NOS (eNOS) signaling which increases NO production or cellular-enzymatic antioxidant defense (Meng et al., 2009; Park et al., 2015; Li T. et al., 2017). Furthermore, resveratrol can downregulate the expression of different enzyme-generating ROS products such as nicotinamide adenine dinucleotide phosphate (NADPH) oxidase 1 (NOX1), NOX2, NOX4, p22phox, and p47phox as well the NOX complex activity (Csiszar et al., 2006; Addabbo et al., 2009). Moreover, *in vitro* studies demonstrated that resveratrol also reduces the oxidative stress in endothelial progenitor cells (EPCs) and prevents their apoptosis through peroxisome proliferator-activated receptor (PPAR)-gamma/heme oxygenase-1 (HO-1) pathways (Shen et al., 2016). Similar results demonstrated that resveratrol can inhibit ROS-induced cell death by stimulating AMP-activated protein kinase (AMPK)/sirtuin 1 (SIRT1)/peroxisome proliferator-activated receptor-gamma coactivator-1 (PGC-1) alpha pathway (Li et al., 2017a; Huang et al., 2021). Recent studies have demonstrated that resveratrol can exert its cardioprotective role through the modulation of the SIRT1/c-Jun N-terminal kinase (c-JNK)/p53 pathway (Ibrahim et al., 2022) or through the indirect activation of SIRT1 modulating different pathways such as the inhibition of phosphodiesterase (PDE) and subsequent elevation of cellular nicotinamide adenine dinucleotide (NAD^+^) (Park et al., 2012), by enhancing the binding of SIRT1 to lamin A (Liu et al., 2012) or by the upregulation of SIRT1 expression (Csiszar et al., 2009; Xia et al., 2013). Resveratrol can directly interact with SIRT1 (Howitz et al., 2003; Hubbard et al., 2013) as well as increase its activity by rising the intracellular NAD + concentration, which is dependent on phosphodiesterase (PDE) inhibition, leading to the phosphorylation of AMPK (Park et al., 2012), or enhancing the binding of SIRT1 to lamin A (Liu et al., 2012; Park et al., 2012; Alexander et al., 2015). AMPK can also be activated by resveratrol and other polyphenols likely through the inhibition of mitochondrial ATP generation (Zheng and Ramirez, 2000). AMPK leads also to an increase in cellular NAD levels indirectly stimulating SIRT1, which utilizes NAD as a substrate (Cantó et al., 2009). Furthermore, SIRT1 activation can protect cells against oxidative stress through its deacetylating activity on different transcription factors that control the expression of many genes, such as superoxide dismutase 2 (SOD2) (Milne and Denu, 2008). SIRT1 activation led also to the downregulation of thrombosis-related markers P-selectin, P-selectin glycoprotein ligand 1 (PSGL-1), and Von Willebrand factor (vWF) (Lou et al., 2017). Another target of resveratrol is Nrf2 that, after nucleus translocation, binds to the promoter sequence of antioxidant response element (ARE) and controls the expression of different antioxidant enzymes including glutathione reductase and HO-1 (Ungvari et al., 2010; Kweider et al., 2014; Xia et al., 2014). At the endothelial level, resveratrol can increase NO production through different mechanisms that can include the prevention of NO degradation (Morrison and Pollock, 1990), the upregulation of endothelial NO synthase (eNOS), the enhancement of eNOS activity or the prevention of eNOS uncoupling (Xia et al., 2014). The interaction between resveratrol and SIRT1 leads to the activation of Forkhead box O (FOXO) factors, downstream targets of SIRT1, which in turn can upregulate the expression of eNOS (Xia et al., 2013). The activation of SIRT1 induces an upregulation of GTP cyclohydrolase 1 (GCH1) increasing the biosynthesis of tetrahydrobiopterin (BH4) (Li et al., 2019) which is a eNOS cofactor that prevents eNOS uncoupling (Förstermann and Münzel, 2006; Li and Förstermann, 2013; Förstermann et al., 2017). Furthermore, resveratrol seems to increase eNOS phosphorylation *in vitro* (Klinge et al., 2005; 2008) leading to an increased activity of this enzyme with consequently increased NO production (Fleming, 2010; Heiss and Dirsch, 2014). Additionally, resveratrol increases eNOS activity by inducing SIRT1-mediated deacetylation of eNOS and by upregulating the enzyme dimethylarginine dimethylaminohydrolase (DDAH) that is involved in the degradation of the eNOS inhibitor asymmetric dimethylarginine (ADMA) (Maas et al., 2009; Frombaum et al., 2011). Resveratrol also exhibits anti-inflammatory properties. Endothelial cells acquire two activated phenotypes during the inflammatory process. Type 1 phenotype is a rapid and transitory response while type 2 phenotype is a steady response that promotes the expression of inflammatory cytokines and adhesion molecules (Gimbrone and García-Cardeña, 2016). Lysophosphatidylcholine (LPC) is thought to be associated with coronary artery inflammation and the increase of pro-inflammatory cytokines (Strowig et al., 2012) that could be inhibited by resveratrol through Toll-like receptor-4 (TLR-4)/Myeloid differentiation primary response 88 (MyD88)/NF-kB signaling pathways (Sheldon et al., 2014; Yanez et al., 2019). Resveratrol could increase the expression of Krüppel-like factor-2 (KLF2), involved in the prevention of atherosclerosis, which led to a reduction in pro-inflammatory cytokines (Chu et al., 2018) and various adhesion molecules including vascular cellular adhesion molecule-1 (VCAM-1), intercellular adhesion molecule 1 (ICAM-1), E selectin, and monocyte chemoattractant protein-1 (MCP-1) (SenBanerjee et al., 2004; Chu et al., 2018). Another mechanism mediated by resveratrol on endothelial cells is the downregulation of endothelin-1 (ET-1), a potent vasoconstrictor (Nicholson et al., 2008) implicated in the development of vascular disease and atherosclerosis (Corder et al., 2001). Furthermore, different studies have demonstrated the effects of resveratrol on vascular remodeling. Smooth muscle cell (SMC) proliferation is essential for the maintenance and repair of the vasculature, on the other hand, excessive proliferation due to vascular injury promotes the development of atherosclerosis, restenosis, and pulmonary hypertension (Thompson et al., 2014; Wang et al., 2018). *In vitro* studies have shown that resveratrol treatment can inhibit SMC proliferation likely through the inhibition of the phosphoinositide 3-kinases (PI3K)/Akt/mTOR pathway (Mnjoyan and Fujise, 2003; Poussier et al., 2005; Brito et al., 2009). Furthermore, resveratrol treatment also prevents arterial stiffness likely by the activation of SIRT1 which exerts anti-inflammatory properties through the inhibition of NF-kB and the downregulation of VCAM-1 and p47phox (Fry et al., 2016).

## 5 Role of resveratrol gut-microbiota derived metabolites on CVD

The human microbiome is the term used for the trillions of microorganisms that cohabit in and on us (Ursell et al., 2012). Microbiome research has surged with remarkable speed in the last 20 years, unveiling the numerous ways in which these tiny inhabitants influence our everyday existence. It has become evident that the microbiota plays a pivotal role in shaping human health, affecting disease outcomes, and governing host physiology (Cryan et al., 2019). The most representative phyla are Firmicutes and Bacteroidetes, followed by Proteobacteria and Actinobacteria (Jin et al., 2019). Among factors influencing microbiota composition, diet plays a pivotal role as it provides the substrates that facilitate the proliferation of specific taxa over others. Clearly, variations in microbiota composition also impact the metabolites produced, which can either positively or negatively influence the host’s health status (Gentile and Weir, 2018; Fan and Pedersen, 2021). Many studies demonstrated that alteration in the gut microbiota composition and relative metabolites are associated with different conditions, such as neurodegenerative disease (Cryan et al., 2019), diabetes (Patterson et al., 2016), cancer (Park et al., 2022) and CVD (Rahman et al., 2022). One of the main metabolites correlated with increased cardiovascular disease was trimethylamine-N-oxide (TMAO). This metabolite is produced in the liver from the microbial-derived trimethylamine (TMA), metabolized by nutrients abundant in the Western diet such as lecithin, choline, and carnitine (Witkowski et al., 2020). Furthermore, the Western diet leads to the proliferation of bacterial species characterized as pro-inflammatory. The establishment of a pro-inflammatory state also results in alterations to the intestinal barrier (leaky gut), promoting the translocation of harmful molecules (Christovich and Luo, 2022) and the establishment of a low-grade chronic inflammatory state (van den Munckhof et al., 2018), one of the main risk factors for different pathologies, including CVD (Munger et al., 1996; Rauchhaus et al., 2000). Various studies have investigated the possible interaction between gut microbiota dysbiosis and CVD. An increase of *Prevotella* and *Klebsiella* genera and a reduction of *Faecalibacterium*, *Oscillibacter*, *Roseburia*, *Bifidobacterium*, *Coprococcus*, and *Butyrivibrio* have been observed in hypertensive and pre-hypertensive participants (Li et al., 2017b). Similarly, decreased abundance of *Faecalibacterium prausnitzii* and Lachnospiraceae family and increased levels of *Ruminococcus*, *Prevotella*, *Hungatella*, and *Succinclasticum* genera were reported for participants with heart failure (Oniszczuk et al., 2021).

In food products, resveratrol is primarily present in its glycosylated form, known as piceid and polydatin (Chaplin et al., 2018). Once ingested, resveratrol travels through the gastrointestinal tract, with an estimated 70% absorption rate (Gambini et al., 2015). Within the intestine, resveratrol binds different nutrients which influence its absorption capacity (Gambini et al., 2015). However, the free form of resveratrol reaches low concentration in the blood as it is metabolized mainly in the liver through processes of glucuronidation and sulfation (Walle, 2011). Resveratrol-3-sulfate and resveratrol-3-glucuronide have been detected in different organs and tissues such as the liver, adipose tissue, and heart (Andres-Lacueva et al., 2012; Bresciani et al., 2014). Moreover, resveratrol could be metabolized in other derivatives, such as piceatannol and dihydroresveratrol (Potter et al., 2002; Menet et al., 2017). Piceatannol is produced through hydroxylation of resveratrol in the liver (Potter et al., 2002), while dihydroresveratrol through the gut bacteria metabolism (Menet et al., 2017). The importance of the gut microbiota in resveratrol metabolism is becoming increasingly evident. In particular, it was observed that gut bacteria can hydrolyze the glucoside form of resveratrol, piceid, producing resveratrol and *vice versa* (Chaplin et al., 2018). *Bifidobacteria infantis* and *Lactobacillus acidophilus* have been identified as bacteria involved in the synthesis of resveratrol from piceid (Wang et al., 2011; Basholli-Salihu et al., 2016; Theilmann et al., 2017). Resveratrol and its precursors could be metabolized by gut microbiota producing resveratrol metabolites. The first resveratrol-derived metabolite identified was dihydroresveratrol, which is produced by *Slackia equolifaciens* and *Adlercreutzia equolifaciens*, followed by 3,4′-dihydroxy-trans-stilbene and 3,4′-dihydroxybibenzyl (lunularin) (Bode et al., 2013). Furthermore, additional studies demonstrated that other bacteria, such as *Bacillus cereus*, *B. infantis,* and *L. acidophilus*, are responsible for piceid production (Cichewicz and Kouzi, 1998; Wang et al., 2011; Basholli-Salihu et al., 2016). Gut bacteria could also metabolize piceid to produce dihydropiceid and dihydroresveratrol (Wang et al., 2011).

Various studies have investigated the role of resveratrol-derived metabolites on cardiovascular outcomes. In an *in vitro* study conducted on isolated rat thoracic aorta, it was evaluated the effects of different metabolites extracted from the rhizome *Rheum undulatum* (Yoo et al., 2007). The extract included seven hydroxystilbene components as active principles (piceatannol, resveratrol, desoxyrhapontigenin, rhapontigenin, piceid, rhaponticin, and ε-viniferin) (Yoo et al., 2007). Of these, piceatannol (a resveratrol metabolite) exhibited the most potent vascular relaxation effect, which was diminished after the removal of functional endothelium or by pretreatment of the aortic tissues with N^G^-nitro-L-arginine methyl ester (L-NAME), a well known non-selective nitric oxide synthase inhibitor (Yoo et al., 2007). Furthermore, *in vivo* piceatannol administration, in a rat model of obesity, tended to reduce the heart/body weight ratio, generally used as a parameter for heart hypertrophy (Hijona et al., 2016). Furthermore, it was observed that the piceatannol at the dose of 45 mg/kg can increase significantly ephrin-B1 protein level, a structural protein essential for cardiac tissue architecture (Hijona et al., 2016). In another *in vivo* study, it was evaluated the effects of resveratrol treatment on atherosclerosis (Chen et al., 2016). Resveratrol supplementation effectively reduced TMA production, and consequently, derived metabolite (TMAO), and regulated bile acid metabolism in both C57BL6J and ApoE −/− mice (Chen et al., 2016) as well as reduced atherosclerotic lesion size, alleviated hyperlipidemia, ameliorated hepatic lipid accumulation, and promoted lipid metabolism in ApoE −/− mice (Cheng et al., 2023). The positive effects of resveratrol supplementation were associated with changes in the microbiota composition with a significant increase in the abundance of *Bacteroides*, *Lactobacillus*, *Bifidobacterium*, *Verrucomicrobia* and *Akkermansia* genus (Chen et al., 2016; Cheng et al., 2023). In line, another experimental study demonstrated that resveratrol supplementation may influence not only gut microbiome but also intestinal integrity biomarkers (Chen et al., 2020). Moreover, resveratrol intake was associated with increased total physical activity and exercise capacity with enhanced skeletal muscle metabolism and function in an animal model of heart failure (Sung et al., 2017). In another study, fecal transplantation from resveratrol-fed mice donor to recipient mice was associated with improved glucose homeostasis and decreased colon inflammation which was also associated with reduced blood pressure after angiotensin-II infusion (Kim et al., 2018). Furthermore, it was demonstrated that sterile fecal filtered from resveratrol-fed mice was sufficient to improve glucose homeostasis in obese mice (Kim et al., 2018). Animal models of high-fructose diet during pregnancy and lactation are used to study the hypertension development in offspring (Tain et al., 2018). Many studies investigated the effects of resveratrol administration during pregnancy and lactation on offspring outcomes. Maternal resveratrol supplementation during pregnancy and post-weaning was shown to exert beneficial effects on offspring reducing renal oxidative stress, restoring mRNA levels of genes involved in the nutrient-sensing pathways *Prkaa2*, *Prkag2*, *Ppara*, *Pparb*, *Ppargc1a*, and *Sirt4* and prevent hypertension associated with high-fructose intake modulating the gut microbiota composition and restoring the *Firmicutes* to *Proteobacteria* ratio (Tain et al., 2018). Similar results demonstrated that resveratrol administration can protect male offspring from hypertension accompanied by a significant downregulation of angiotensinogen, renin, prorenin receptor, angiotensin-converting enzyme (ACE), angiotensin II type 1 receptor (AT1R), but increased ACE2, angiotensin II type 2 receptor (AT2R) and angiotensin (1–7) receptor MAS (Hsu et al., 2021). The beneficial effects of resveratrol supplementation were associated with changes in the microbiota composition with increased abundance of butyrate-producing genera *Akkermansia*, Lachnospiraceae and Ruminococcaceae, as well as Cyanobiaceae and Erysipelotrichaceae family (Hsu et al., 2021). Concerning short-chain fatty acid (SCFA)-producing bacteria, different studies demonstrated that resveratrol administration can increase the abundance of *Allobaculum*, *Bacteroides* and *Blautia* (Alrafas et al., 2019; Wang et al., 2020). SCFAs, particularly butyrate, are well known to be inhibitors of histone deacetylase (HDAC). The protective effects exerted by SCFAs are likely mediated by their HDAC inhibitory activity on intestinal macrophages resulting in the suppression of proinflammatory cytokines production (Evans et al., 2020). Furthermore, SCFAs have been demonstrated to attenuate cardiac hypertrophy, fibrosis, and dysfunction in various animal models of CVD (Chen et al., 2015; Zhang et al., 2017; Patel, 2018). SCFAs also showed the ability to regulate blood pressure through the interaction with two receptors, the Olfactory receptor 78 (Olfr78) and the G protein-coupled receptor 41 (Gpr41), both expressed in smooth muscle cells of blood vessels (Pluznick, 2014; Miyamoto et al., 2016).

## 6 Conclusion

The findings from clinical studies on the effects of resveratrol on cardiovascular disease are difficult to interpret because the effects on both vascular and endothelial outcomes are inconsistent, and rather unrelated to the dose. In fact, most studies supplementing red wine, even dealcoholized type (to eliminate the potential confounding effect of alcohol) resulted in significant effects although the resveratrol content was, on average, much lower than tablet supplementation (about 1–3 mg/day vs. 100–300 mg/day), while other studies with often extreme resveratrol supplementation (i.e., >500 mg/day) resulted in null findings. The conflicting findings from RCTs could be potentially explained through the differences in the real exposure to resveratrol metabolites, in part attributed to the interindividual variations in the physiological response to resveratrol intake due to differences in gut microbiota composition. Additionally, the potential interactions, including accumulating, synergistic, and antagonistic effects, with other food matrix components cannot be ruled out. Many *in vitro* and *in vivo* studies suggested that resveratrol cardioprotective effects are mediated by the activation of different antioxidant, anti-inflammatory, and anti-hypertensive pathways. These are also accompanied by changes in microbiota composition. In particular, most of the studies agreed with the increase in the abundance of SCFA-producing bacteria. SCFAs showed both direct and indirect cardioprotective effects through the attenuation of cardiac dysfunction and modulation of the inflammatory state. For these reasons, it is important to further explore the role of the gut microbiota in modulating the effects of resveratrol supplementation and its effects in preventing cardiovascular pathologies.
